# Pulmonary Rehabilitation in Patients with Operable Non-Small Cell Lung Cancer

**DOI:** 10.3390/jcm14030770

**Published:** 2025-01-24

**Authors:** Jeffrey Zhong, Ilene Trinh, Shine Raju, Melinda Hsu

**Affiliations:** 1 Case Western Reserve University School of Medicine, Cleveland, OH 44106, USA; jyz7@case.edu (J.Z.); ixt86@case.edu (I.T.); shine.kochukunjuraju@uhhospitals.org (S.R.); 2 Department of Pulmonary, Critical Care, and Sleep Medicine, University Hospitals Cleveland Medical Center, Cleveland, OH 44106, USA; 3 Department of Hematology and Oncology, University Hospitals Seidman Cancer Center, Case Western Reserve University, 11100 Euclid Ave., Cleveland, OH 44106, USA

**Keywords:** pulmonary rehabilitation, lung cancer survivorship

## Abstract

Lung cancer is the leading cause of cancer-related death worldwide, and patients with operable early-stage NSCLC are typically managed surgically. While effective, surgical resection can significantly impact pulmonary function and quality of life. Pulmonary rehabilitation (PR) is a comprehensive, multimodal approach that is an established cornerstone in the treatment of COPD. It has similarly demonstrated multiple benefits in patients with lung cancer who have undergone lobectomy or resection by improving pulmonary function, increasing exercise tolerance, improving nutritional status, providing psychological support, and enhancing quality of life. Despite this, PR for early-stage operable NSCLC is oftentimes not standardized, and challenges to adherence remain. In this review, we examine the components of PR, the role of PR in pre- and postoperative settings in patients with early-stage NSCLC, implementation strategies for PR, and future directions and challenges of PR in operable NSCLC.

## 1. Introduction

Lung cancer is the third most common type of cancer and the leading cause of cancer death worldwide [1,2]. Non-small cell lung cancer (NSCLC) accounts for approximately 85% of all lung cancer cases globally, with 30% of patients with NSCLC diagnosed at early stages (stage I or II) [3,4]. Early-stage NSCLC is typically managed through surgical resection and systematic mediastinal lymph node dissection as the standard of care [5,6]. Lobectomy is the gold standard, while segmentectomy and wedge resection are viable alternatives for patients with smaller tumors, limited pulmonary reserve, inability to tolerate extensive surgical procedures, or contraindications to lobectomy [6,7]. Patients who are not surgical candidates or have poor pulmonary reserve may undergo definitive radiotherapy [8].

Following surgical management of early-stage NSCLC, addressing respiratory function is crucial due to the significant impact that lobectomies can have on pulmonary function and quality of life. Lobectomies can result in a marked reduction in lung volumes and respiratory muscle strength, predisposing patients to develop respiratory failure, which can prolong hospitalization, increase healthcare costs, decrease quality of life, and increase mortality [9,10]. Up to 8% of patients who had undergone lobectomy for early-stage lung cancer developed respiratory failure while hospitalized [9]. Thus, addressing and identifying strategies for improving respiratory function following surgical management of early-stage lung cancer is imperative.

Pulmonary rehabilitation (PR) aims to improve pulmonary function and quality of life in patients with chronic respiratory diseases, and it can also be utilized post-surgery to similarly improve respiratory function [11]. PR is a broad, adjunct therapy that encompasses exercise training, education, healthy behavior change, and psychological support [12,13]. The role of PR has been established in stable chronic obstructive pulmonary disease (COPD) and following hospitalization for COPD exacerbations as standard of care, with improvements in symptoms including dyspnea, fatigue, exercise tolerance, and quality of life [14,15,16,17]. PR has also been shown to reduce hospitalizations for COPD and improve mortality after hospitalizations [18,19].

The role of PR following surgical resection of early-stage NSCLC remains less established than in chronic respiratory diseases. In this review, we will evaluate and review the evidence of PR in NSCLC surgery patients in the pre- and postoperative settings.

## 2. The Components and Mechanisms of Pulmonary Rehabilitation

### 2.1. Key Components of Pulmonary Rehabilitation

**Figure 1:**
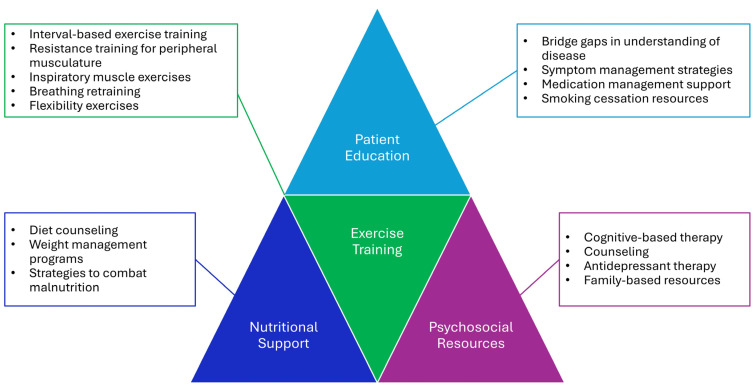
Components and interventions of pulmonary rehabilitation.

PR is a comprehensive, multidisciplinary intervention involving thorough patient evaluation and patient-tailored therapies such as exercise training, education with promotion of healthy behaviors, nutritional support, and psychosocial care [12,13]. Specific components and interventions of PR are detailed in Figure 1. These interventions aim to reduce symptoms, optimize functional status, lower hospitalization rates, and decrease healthcare costs [13]. Contraindications to PR include conditions that can place patients at increased risk during exercise or present as obstacles to participation. These can include unstable cardiac disease, severe orthopedic issues, significant cognitive impairment, unmanaged pain, and hypoxemia refractory to correction with supplemental oxygen [11,20].

Exercise training, a cornerstone of PR, primarily involves endurance training, with interval and resistance training serving as alternatives for patients with lower exercise tolerance. Endurance training primarily consists of walking, cycling, and treadmill exercises [17]. For patients who are not able to tolerate continuous high-intensity exercise, interval training with alternating periods of high- and low-intensity exercise can be effective [21,22,23,24]. Resistance training helps to strengthen major muscle groups in the upper and lower extremities through targeted weightlifting or resistance band training [17]. Similar to interval training, resistance training may be more tolerated than endurance training as it involves lower oxygen ventilation requirements.

Alternative methods of exercise training include inspiratory muscle training, breathing retraining, and flexibility exercises. Inspiratory muscle training (IMT) focuses on strengthening the ventilatory muscles, with a meta-analysis of 32 randomized controlled trials showing that the addition of IMT to general exercise training programs in PR resulted in significant improvements in maximal inspiratory pressure and exercise performance in patients with COPD [25]. Breathing retraining aims to decrease the effects of rapid shallow breathing patterns with diaphragmatic breathing, pursed-lip breathing, and yoga breathing [26,27].

Education is another cornerstone of PR that aims to promote effective self-management of respiratory disease. The components of education in PR are broad but include pathophysiology of disease, breathing training strategies, medication management, symptom management, smoking cessation, health preservation, and the importance of exercise [28].

Nutritional counseling and weight management help to address the obesity, malnutrition, and nutrient deficiencies that patients with decreased respiratory function are at greater risk for. Nutritional interventions, such as patient-tailored counseling and nutritional therapy, can improve body weight status, quality of life, and exercise capacity [29].

Psychological interventions including cognitive behavioral therapy (CBT), psychosocial counseling, and short courses of antidepressant medications can help to mitigate anxiety and depression commonly seen in pulmonary disease patients. Additionally, integrating psychological support into PR can improve patient adherence and completion rates, as depression and anxiety can be significant predictors of PR dropout [30]. Holistic approaches with family-based psychosocial support have also shown promise in enhancing coping and psychosocial adjustments to illness in the family system [31].

### 2.2. Mechanism of Benefit

PR enhances respiratory physiological function through multiple mechanisms. PR directly strengthens inspiratory muscles, resulting in improved respiratory mechanics [26]. Exercise training involved in PR can also induce skeletal muscle adaptations such as increased capillary density, blood flow, and mitochondrial volume density [26,32]. A retrospective study by Choi et al. found better preserved Hounsfield units of erector spinae muscle (*p* = 0.001) and decreased muscle loss (0.6% vs. 3.4%; *p* = 0.003) in patients with NSCLC who had undergone lung cancer surgery that had received pre- and postoperative PR than those who had not undergone PR [33]. Decreased levels of lactic acidosis from peripheral skeletal muscles help offset the burden of increased respiratory drive on respiratory muscles [32]. Decreased ventilatory requirements during physical activities can also mitigate the increased work of breathing post-lobectomy and thereby improve overall lung function [26,32]. With continued adherence to PR, enhancements in exercise tolerance are shown by improvements in the 6-min walk test (6MWT) and incremental shuttle walk distance (ISWD) [14]. Furthermore, PR in post-lobectomy patients has been shown to improve and mitigate declines in forced expiratory volume (FEV1) and vital capacity (VC) [34].

Additionally, PR improves the psychological and emotional status of patients through reductions in anxiety and depression, enhancing coping strategies, and fostering a sense of empowerment [35,36,37]. A systematic review of 29 randomized control trials of psychological and lifestyle interventions noted significant improvements in symptoms of anxiety and depression in interventions that included an exercise component [38]. The addition of CBT into PR has also demonstrated improvements in fatigue, stress, anxiety, and depression, as patients are able to modify negative thought patterns and behaviors associated with their illness [35,39]. Education of coping strategies and self-management strategies with relaxation techniques can also help patients manage stress and anxiety more effectively [40]. Furthermore, a retrospective cohort study found that 88% of patients with loss of dignity who received interdisciplinary PR no longer reported significant issues with respect to loss of dignity [41].

## 3. Current Evidence of Pulmonary Rehabilitation and Lung Cancer Surgery

### 3.1. Preoperative Pulmonary Rehabilitation and Surgical Management of NSCLC

PR in patients with early-stage NSCLC undergoing surgery can be useful in both the preoperative and postoperative settings (Table 1). Many patients with resectable NSCLC are deemed inoperable due to inadequate pulmonary function [42]. Preoperative PR can improve pulmonary function for patients to achieve eligibility for surgery. This is especially beneficial for patients with NSCLC and concurrent COPD, which PR has traditionally been used for. Some studies have shown that preoperative PR can improve pulmonary function enough to allow initially inoperable patients to undergo surgery [43,44,45]. For example, in a study by Goldsmith et al., 58.8% of lung cancer patients in their cohort were initially considered inoperable. After preoperative rehabilitation, however, 75.8% were successfully deemed operable, demonstrating the utility of PR in improving rates of eligibility for surgery in lung cancer patients [43]. In another study by Pehlivan et al., 60% of patients with lung cancer initially considered inoperable were able to have surgery after 15 days of preoperative PR [45].

Preoperative PR can also be useful in improving postoperative outcomes in patients undergoing surgery. A study by Lai et al. found that patients enrolled in an intensive seven-day PR preoperative program had reduced hospital length of stay as well as postoperative pulmonary complications compared to the control group [46]. Additionally, Saito et al. reported that involving home-based PR demonstrated a reduction in postoperative complications, although no effect was seen on hospital length of stay [47]. Another study by Bobbio et al. in patients with NSCLC and COPD undergoing surgical resection found significant improvement in exercise capacity and an increase in maximal oxygen consumption of 2.8 mL/kg/min following surgery [42]. Concerns regarding preoperative PR delaying cancer-directed treatment have been allayed by multiple studies, finding that a delay of less than one month does not have a significant impact on patient outcomes [42,61,62]. Furthermore, preoperative PR has been found to improve symptom burden in patients with lung cancer. Analysis by Mujovic et al. demonstrated that 2–4 weeks of preoperative PR among patients with NSCLC and COPD significantly improved performance on 6MWT after surgery [48]. Patient-reported dyspnea was also reduced following preoperative PR but was not significantly changed after surgery [46]. Similar results of decreased dyspnea and improved performance on 6MWT were reported in the study by Goldsmith et al. [43].

### 3.2. Postoperative Pulmonary Rehabilitation Following Surgical Management of NSCLC

The utilization of PR adjuvantly in patients with lung cancer has been shown to be an effective means of improving pulmonary function and QoL. Tao et al. examined the impact of personalized PR exercise training in addition to inhaled albuterol nebulizer solution versus the control group with inhaled albuterol nebulizer only in 88 patients who had undergone thoracoscopic lobectomy [50]. Forced vital capacity (FVC) (2.51 ± 0.61 L vs. 2.69 ± 0.49 L; *p* < 0.5), FEV1 (1.75 ± 0.53 L vs. 2.27 ± 0.48 L; *p* < 0.5), maximum voluntary ventilation (MVV) (61.44 ± 11.22 L/min vs. 70.19 ± 13.07 L/min), peak expiratory flow (PEF) (4.72 ± 0.53 L/s vs. 5.39 ± 0.61 L/s), and 6MWT (290.44 ± 30.65 m vs. 233.85 ± 24.71 m; *p* < 0.5) were significantly improved in the PR group compared to control at three months follow up. Additionally, Niu et al. observed similar improvements with PR in pulmonary function postoperatively in patients with NSCLC two to three weeks following surgery in a retrospective analysis [51]. Forty-six patients who had received PR following surgery for NSCLC demonstrated improvements in FEV1 (2.31 L/min vs. 1.75 L/min, *p*  <  0.001), predicted FVC (88.75% vs. 68.30%, *p*  <  0.001), and FEV1/FVC (64.17% vs. 50.87%, *p*  <  0.001) in comparison to a propensity score-matched control group of patients who did not receive PR postoperatively at two to three weeks after surgery [51]. Riesenberg and Lubbe conducted a prospective study to assess the changes in exercise capacity and quality of life before and after a 28-day inpatient PR program with standardized aerobic training. Significant improvements in 6WMT (322 ± 11 to 385 ± 13 m, *p* < 0.001) and QoL assessed via QLQ-C30 and QLQ-LC13 (48 ± 3 to 62 ± 2, *p* < 0.001) at 28 days was noted [52].

PR has also been shown to improve symptoms and pulmonary complication rates post-lobectomy for NSCLC. A retrospective study investigating the impact of PR that included 1-week pre- and 3-month postoperative exercise interventions of breathing, flexibility, resistance, aerobic exercise, coughing/huffing techniques, and early mobilization in 41 NSCLC patients who had undergone video-assisted thoracoscopic surgery (VATS) or posterolateral thoracotomy (PLT) found that maximum mouth inspiratory pressure (Pimax) and modified Medical Research Council (mMRC) dyspnea scale improved preoperative values at 3 months post-lobectomy [56]. Another retrospective study examining the effectiveness of a 3-week inpatient PR program in NSCLC postoperative patients noted that patients with COPD after lobectomy due to NSCLC show similar improvements in 6MWT and dyspnea as patients with COPD without lobectomy for NSCLC and patients with lobectomy for NSCLC without COPD [53]. Moreover, a multi-center randomized control trial including a total of 374 patients scheduled for lung surgery with 1:1 randomization to preoperative and postoperative PR in addition to enhanced recovery after surgery (ERAS) protocol versus ERAS protocol alone demonstrated a significantly decreased incidence of postoperative pulmonary complications in the experimental group at 14 weeks (18.7% vs. 33.2%, HR 0.524, 95% CI 0.347 to 0.792, *p* = 0.002) [57].

### 3.3. Multimodal Pulmonary Rehabilitation in Surgical Management of NSCLC

Multimodal interventions, including PR, have resulted in improved functional capacity, exercise tolerance, and nutritional status in patients undergoing curative operations for NSCLC. A randomized control study of 169 patients with pathological diagnosis of stage I or II NSCLC following thoracoscopic lobectomy of lung cancer were stratified to undergo either two-week preoperative PR plus individualized nutrition program with one-to-one nutritional consultation and dietary guidance or no PR or an individualized nutrition program as the control group [58]. The cohort that received PR and an individualized nutrition program exhibited significant improvements in 6MWT, BMI, total protein, albumin, and hemoglobin levels at 4, 8, and 12 weeks postop in the experimental group. Another randomized control trial consisting of 73 patients with stage I–III NSCLC scheduled for VATS lobectomy demonstrated increased efficacy of a 2-week preoperative multimodal PR program (exercise training, nutritional counseling with whey protein supplementation, and psychological interventions) compared to usual clinical care with an FVC of 0.35 L greater (95% CI 0.05–0.66; *p* = 0.021) and a 6MWT of 60.9 m greater (95% CI 32.4–89.5; *p* < 0.001) in comparison to the control group receiving usual clinical care [49].

There is also evidence that psychological interventions may improve the psychological status of postoperative NSCLC patients. An exploratory trial including 28 post-surgical NSCLC patients was randomized to undergo a 3-month self-efficacy enhancing intervention based on motivational interviewing in addition to PR versus routine care with standard PR [59]. Overall, the self-efficacy-enhancing intervention group was superior to the control group in reducing symptoms of anxiety and depression, confrontational coping, and improving self-efficacy. Wang et al. showed that cognitive behavioral stress management in postoperative NSCLC patients significantly improved hospital anxiety and depression scale (HADS) scores at 3 months and 6 months, with significant decreases in depression severity at 6 months [54]. Another randomized clinical trial consisting of 24 patients with NSCLC eligible for lung resection who underwent 4 weeks of PR versus chest physical therapy noted improved levels of anxiety and depression based on HADS scores [60]. Though the evidence of the psychological benefits of multimodal PR is limited in the postoperative NSCLC setting, there are promising results of different psychological interventions that can be incorporated into PR. The evidence of improvements in psychological status in the setting of COPD is more established [38], and there are likely additional psychological benefits that have yet to be described in PR in lung cancer.

## 4. Implementation Strategies for Pulmonary Rehabilitation in Lung Cancer

### 4.1. Design of PR Programs Specific to Lung Cancer Patients

The impacts of lung cancer are complex and variable, depending on the patient’s stage of cancer, symptom burden, and treatment regimen. Individualizing rehabilitation is necessary based on specific patient needs and may also be a strategy to increase patient adherence [63]. Support from a multidisciplinary team including but not limited to pulmonologists, thoracic surgeons, nurses, respiratory therapists, physical therapists, dietitians, and social workers is necessary to implement all components of an effective PR program for patients with lung cancer.

Previously described basic components of PR include exercise training, breathing training, including respiratory muscle exercises, and education sessions. As a majority of patients with lung cancer are physically inactive due to symptoms, such as cachexia, fatigue, weakness, pain, and dyspnea, exercise training of peripheral muscles is especially important for reconditioning [64,65]. Exercise training should include both aerobic and resistance training. Aerobic exercise can be incorporated through cycling or treadmill exercises of 30 min sessions starting at low-intensity activity (50% of maximum heart rate) with incremental progression to moderate- or high-intensity activity (80% of maximum heart rate), which is a common regimen in other successful programs [42,44,55,66]. Resistance training should include exercises for both upper and lower extremity conditioning given the high rates of cachexia in patients with lung cancer [67].

PR can be delivered in a variety of modalities, including inpatient, outpatient, home based, or a hybrid of these options. Site-based programs, such as inpatient and outpatient programs, are beneficial for the standardization of care and the camaraderie of group-based care. Home-based PR programs minimize transportation barriers and decrease time toxicity. However, home-based PR lacks the community aspect of traditional site-based programs and may result in lower rates of patient adherence, partly attributable to the absence of peer pressure from a group setting [68]. Additionally, some studies on home-based PR programs have suggested that the impact on patient outcomes may be limited compared to site-based programs. Although the study by Saito et al. demonstrated a reduction in postoperative complications in a preoperative PR program, no impacts on the length of hospital stay were seen [69]. This differs from other data that show preoperative PR can reduce the length of hospital stay and may be attributed to the difference in mode of delivery as a home-based program. Given the unique needs of patients with varying stages of lung cancer, as well as the busy schedule of lung cancer patients with many medical appointments, we recommend tailoring the modality of PR based on the patient’s individualized needs and circumstances.

Another pertinent aspect to consider for optimizing PR for lung cancer patients is addressing the psychological and emotional needs of the patient. As lung cancer patients are at particularly high risk of emotional distress and symptom burden compared to other cancer patients [65,70,71], utilizing PR for its psychological benefits may be just as important as its physical benefits. One of the reasons PR is effective in providing psychological benefits is the inclusion of group therapy, which provides a sense of community. Patients in exercise intervention programs have reported significant benefits from the social support that group sessions provide [66,68]. Therefore, no matter which modality is chosen for PR, it is essential to incorporate a community component to some extent.

### 4.2. Timing and Delivery of PR

For preoperative PR, we suggest a short-term intensive program in the perioperative setting of up to four weeks of therapy to minimize delay in necessary treatment. The duration of preoperative PR can be tailored to the patient’s individualized goals and needs. For example, patients seeking PR due to inoperability may require a longer duration compared to patients pursuing PR only for conditioning. Lai et al. indicated that an intensive PR program of as short as seven days was able to impact postoperative outcomes [46]. This suggests that a patient’s PR program can be tailored to as short as one week with resulting benefits if circumstances are time sensitive.

For postoperative PR, we recommend at least two weeks after surgery to allow for adequate recovery time from surgery and postoperative complications. The findings from a meta-analysis by Sommer et al. suggest that postoperative rehabilitation programs with later initiation (at least two weeks after surgery) may have better outcomes than those with earlier initiation (less than two weeks after surgery) [72]. Another study by Missel et al. reported that the initiation of a postoperative exercise program had a median start time of 15 days following surgery due to postoperative complications [66]. Therefore, it is important to allow for ample recovery time before initiating PR, which is estimated to be at least two weeks. The duration of postoperative PR programs in previous studies demonstrating significant benefits have typically ranged between 4 and 12 weeks with varied settings and intensity levels. However, there is not yet definitive evidence on the optimal regimen for postoperative PR. We suggest tailoring duration based on the patient’s individualized care plan and mode of delivery.

### 4.3. Challenges and Barriers to Effective Implementation

One of the main challenges to the effective implementation of PR is patient adherence, which can be affected by a variety of physical, social, logistic, and psychological factors. Despite the benefits of exercise training in PR in NSCLC patients following surgery, some patients may not be engaged in adequate amounts of physical exercise. Krebs et al. showed that early-stage NSCLC patients were generally adherent to medical health promotion recommendations, but few engaged in the recommended levels of physical activity set by the US Department of Health and Human Services [73]. The burden of symptoms, side effects of chemotherapy, infections, hospitalizations, and comorbid conditions have been reported as barriers to adherence in studies of PR or exercise intervention programs in patients with lung cancer [66,68]. A qualitative longitudinal feasibility study investigating motivation and barriers to PR exercise in patients with operable NSCLC demonstrated that barriers to adherence were primarily related to the side effects of chemotherapy [66]. Social and logistic reasons such as return to work, medical appointments, social or personal commitments, and cost may also contribute to nonadherence [66,68]. Barriers to attendance of site-based programs include transportation and scheduling conflicts. Access to inpatient programs is also limited by cost and available beds [64]. It is also notable to consider resource limitation as a significant challenge to establishing PR programs, especially given the role of multidisciplinary staff members in PR. However, to alleviate the resource burden, it may be more feasible to first implement PR programs in existing oncology clinics or to offer home-based services.

In addition, PR cannot be effective without patient motivation and self-discipline, which presents another challenge to PR implementation. A feasibility study of home-based PR in patients with thoracic cancers, including lung cancer, found that approximately 65% of eligible patients refused participation due to a lack of motivation [74]. Strategies to enhance patient motivation to undergo PR include resolving knowledge gaps on the wide-ranging benefits and efficacy of PR and discussing PR as an option soon after diagnosis. A study by Adamsen et al. reported that lung cancer patients who were motivated to undergo an exercise intervention program were often offered the option during a window of time in which they had just received their diagnosis and felt anxious to be proactive in their health [68]. Therefore, there may be utility to initiating discussion of PR early after diagnosis and during initial treatment for better patient responses.

## 5. Future Directions and Research Needs

Although the implementation of PR programs for patients with lung cancer has increased in recent years, further research is needed on long-term outcomes in patients who undergo PR. Many studies have revealed the short-term benefits of PR programs; however, less is known on long-term benefits to lung cancer survivorship. The meta-analysis by Sommer et al. found no improvement in exercise capacity on the long-term follow up of over 6 months compared to the short-term follow up of 12 weeks or less across four randomized controlled trials with 262 patients [72]. However, data on long-term follow up were only provided from a single study [72]. Therefore, additional research on long-term outcomes is necessary to better understand the impact of PR on lung cancer survivors.

Additionally, despite increased data on the use of PR in lung cancer management, evidence is mixed due to variety in program modality, intensity, duration, and time to initiation across different studies. Standardization of PR in the preoperative and postoperative settings is necessary to better understand the efficacy of PR on long-term lung cancer patient outcomes and to achieve consistent results in patients undergoing PR. Further prospective studies on controlled trials are needed to determine the optimal regimen, duration, and timing of PR programs for maximal benefit.

Finally, telemedicine is another way to broadly implement PR and further increase access. With the rise of telemedicine use since the COVID-19 pandemic, telemedicine has been used successfully in many rehabilitation settings [75,76,77,78]. Telemedicine can be a useful tool for home-based PR to maintain contact with multidisciplinary personnel and preserve the community component through group calls. A study by Ha et al. demonstrated that telemedicine-based exercise training resulted in an improvement in health-related quality of life in lung cancer patients [79]. However, additional research is needed to assess the impact of telemedicine-based rehabilitation on pulmonary function and endurance capacity in lung cancer survivors. Furthermore, it is important to consider the limitations of technology-based PR, such as digital literacy, access to technology, and the need for family support in utilizing technology, especially for elderly patients in the lung cancer community [12]. Other modes of technology-enhanced rehabilitation with potential for exploration are mobile-app-based and virtual-reality-based programs. Studies in South Korea have trialed mobile-app-based programs with significant improvement in exercise capacity among lung cancer patients [80,81]. Multiple studies have also explored the benefit of virtual-reality-based programs in lung cancer patients [82,83]. A meta-analysis of six randomized controlled trials investigating the efficacy of incorporating VR into PR for patients with COPD noted that VR enhanced the therapeutic effects of PR with significant improvements in FEV1 (*p* < 0.05) and marginally non-significant improvements in 6MWT (*p* = 0.05) [84]. While the utility of VR in PR has not been as well studied in patients with operable NSCLC, a similar approach may prove to be beneficial. Further controlled trials are needed to examine the benefit of technology use in PR for lung cancer patients.

## 6. Conclusions

Although traditionally established for patients with COPD, the benefits of PR are beginning to be explored for patients with lung cancer, with promising results thus far. PR is especially relevant in the management of patients with NSCLC undergoing surgical resection as it can optimize postoperative outcomes and increase rates of eligibility for surgery. Therefore, we stress the importance of incorporating PR into standard of care management for lung cancer patients. With diverse modes of delivery available, the implementation of PR can be flexible to suit each patient’s individualized needs with widespread benefits, ranging from improvement in pulmonary function, exercise capacity, mental health, and overall quality of life. We encourage increased discussion of PR among both clinicians and lung cancer patients to increase awareness of the positive impacts of PR in lung cancer management and urge more focus on research in this field.

## Figures and Tables

**Table 1:** Current evidence of benefits of pulmonary rehabilitation in patients with operable NSCLC.

Reference	Study Type	Duration of Intervention/Assessment	Intervention Description	Primary Outcomes	Secondary Outcomes	Benefits of Intervention
Preoperative setting						
Goldsmith 2020 et al. [43]	Prospective cohort	2–4 weeks	Exercise training, breathing retraining, inspiratory muscle training, education, bronchodilator treatment if required	MRC dyspnea score, performance status, level of activity, 6MWT, frailty index	Postoperative length of hospital stay, complications, and mortality, DLCO, FEV1	Improvement in dyspnea score (*p* = 0.00002), 6MWT (*p* = 0.04), performance status (*p* = 0.003), level of activity (*p* < 0.00001), and frailty index (*p* = 0.0006)
Cesario 2007 et al. [44]	Pilot clinical trial	4 weeks	Exercise training, breathing retraining, inspiratory muscle training education	6MWT, FVC, FEV1, PaO_2_	-	Improvement in FVC (*p* < 0.01), FEV1 (*p* < 0.05), 6MWT (*p* < 0.05), PaO_2_ (*p* < 0.01)
Pehlivan 2019 et al. [45]	Prospective observational	2 weeks	Exercise training, breathing retraining, inspiratory muscle training	FEV1, FVC, mMRC dyspnea score, 6MWT, maximal inspiratory and expiratory pressures for respiratory muscle strength, VO_2_ max	-	Improvement in 6MWT (*p* < 0.001), dyspnea (*p* < 0.001), maximal inspiratory pressure (*p* < 0.001), FVC (*p* < 0.001), FEV1 (*p* = 0.001), and VO_2_ max (*p* < 0.001)
Lai 2007 et al. [46]	Randomized controlled trial	1 week	Exercise training, inspiratory muscle training	6MWT, PEF, quality of life	Postoperative pulmonary complications, postoperative length of stay, total hospital stay	Improved 6MWT (*p* = 0.029) and PEF (*p* < 0.001), decreased postoperative length of stay (*p* = 0.010), total hospital stay (*p* = 0.012), and postoperative pulmonary complication (*p* = 0.037)
Saito 2021 et al. [47]	Retrospective cohort	2–4 weeks	Exercise training, inspiratory muscle training	Postoperative complications	Length of hospital stay, duration of intercostal catheterization	Reduced postoperative complication (*p* = 0.04)
Bobbio 2008 et al. [42]	Prospective observational	4 weeks	Exercise training, breathing retraining	FEV1, TLC, DLCO, VO_2_ max	Workload, oxygen pulse, minute ventilation, breathing reserve, VE/VCO_2_	Improvement in VO_2_ max (*p* < 0.01)
Mujovic 2014 et al. [48]	Prospective observational	2–4 weeks	Exercise training, inspiratory muscle training, education, bronchodilator treatment	6MWT, FEV1, VLC, FEF_50_	Borg dyspnea scale, hospital length of stay, perioperative complications	Improvements in FEV1 (*p* < 0.001), VLC (*p* < 0.001), FEF_50_ (*p* = 0.003), 6MWT (*p* = 0.001)
Liu 2020 et al. [49]	Randomized controlled trial	2 weeks	Exercise training, inspiratory muscle training, nutritional counseling, education	6MWT	PF, length of stay, disability and psychological assessment, short-term recovery quality, PPC, mortality	Improvements in 6MWT (*p* < 0.001), FVC (*p* = 0.21)
Postoperative setting						
Tao 2024 et al. [50]	Randomized controlled trial	3 months	Exercise training, breathing retraining, inspiratory muscle training	FVC, FEV1, MVV, PEF	6MWT, dyspnea index, MOT, length of hospital stay, postoperative pulmonary infections	Improvements in FVC (*p* < 0.05), FEV1 (*p* < 0.05), MVV (*p* < 0.05), PEF (*p* < 0.05), 6MWT (*p* < 0.05), dyspnea index (*p* < 0.05)
Niu 2024 et al. [51]	Retrospective cohort	2–3 weeks	Exercise training, breathing retraining, inspiratory muscle training, education	FEV1, FVC, FEV1/FV, MIP, MEP, cardiopulmonary exercise testing	Cardiac performance assessment, health-related quality of life assessment, muscle measurements	Improvements in FEV1 (*p* < 0.001), FVC (*p* < 0.001), FEV1/FVC (*p* < 0.001), CI (*p* < 0.001), WR (*p* = 0.017), CAT scores (*p* < 0.001)
Riesenberg 2010 et al. [52]	Prospective observational	28 days	Exercise training	FEV1, FVC, 6MWT, work performance via bicycle ergometry	EORTC QLQ-C30, SF-36, MFI-20, HRV	Improvements in work performance (*p* < 0.001), 6MWT (*p* < 0.001), QoL (*p* < 0.001), and fatigue (*p* < 0.001)
Klimczak 2021 et al. [53]	Retrospective cohort	3 weeks	Exercise training, breathing retraining, education, psychological support, nutritional consulting	FEV1, FVC, 6MWT, SGRQ	-	Improvements in 6MWT (*p* < 0.001), SGRQ (*p* < 0.01)
Wang 2023 et al. [54]	Randomized controlled trial	6 months	Cognitive behavioral stress management in addition to usual care	HADS, EORTC QLQ-C30	-	Improvements in HADS depression score (*p* = 0.035), HADS anxiety score (*p* = 0.018), and EORTC QLQ-C30 (*p* < 0.05)
Sterzi 2013 et al. [55]	Prospective observational	3 weeks	Postoperative PR	FEV1, FVC, FEF_25–75%_, pH, O_2_, CO_2_, 6MWT	-	Improvement in 6MWT
Preoperative and postoperative settings						
Ichikawa 2022 et al. [56]	Retrospective observational	1 and 3 months	Exercise training	FEV1, FVC, MIP, MEP, 6MWT	QF, mMRC dyspnea scale	Improvements in 6MWT (*p* < 0.05), MIP (*p* < 0.05), MEP (*p* < 0.05), mMRC dyspnea scale (*p* < 0.05)
Zheng 2023 et al. [57]	Randomized controlled trial	2 weeks	Exercise training, breathing retraining, education	Incidence of PPC	Occurrence of specific complications, time to removal of chest drain, length of hospital stay	Improvements in incidence of PPC (*p* = 0.002)
Li 2024 et al. [58]	Randomized controlled trial	12 weeks	Exercise training, breathing retraining, nutritional support program	FACT-L	PF, 6MWT, BMI, serum total protein, albumin, hemoglobin, incidence of PPC, length of hospital stay, hospitalization costs	Improvements in FACT-L (*p* < 0.001), PF (*p* < 0.001), 6MWT (*p* < 0.001), BMI (*p* < 0.001), serum total protein (*p* < 0.001), albumin (*p* < 0.001), hemoglobin (*p* < 0.001), incidence of PPC (*p* < 0.05), length of hospital stay (*p* < 0.001), hospitalization costs (*p* < 0.001)
Huang 2018 et al. [59]	Randomized controlled trial	3 months	Inspiratory muscle training, education, self-efficacy enhancing intervention based on motivational interviewing	Feasibility (assessed via rates of recruitment, adherence, and retention), and acceptability (assessed with a five-point Likert scale and semi-structure interview)	SESPRM-LC, HADS, social support, subjective well-being, coping styles, posttraumatic growth inventory, BODE index, PF	Improvements in SESPRM-LC, confrontational coping, and social support
Morano 2014 et al. [60]	Randomized controlled trial	4 weeks	Exercise training, inspiratory muscle training, education, nutrition counseling	Serum fibrinogen, serum albumin, PF	6MWT, SF-36, HADS	Improvements in serum fibrinogen (*p* < 0.0001), HADS depression score (*p* = 0.02), HADS anxiety score (*p* = 0.002), physical component of SF-36 (*p* = 0.07)
Choi 2021 et al. [33]	Retrospective cohort	3 weeks	Exercise training, breathing retraining, inspiratory muscle training, education	PF, muscle loss via CT imaging	Major postoperative complications	Improvements in FEV1 (*p* = 0.001), better preserved Hounsfield units of erector spinae muscle (*p* = 0.001), muscle loss (*p* = 0.003), decrease in the incidence of embolic events (*p* = 0.044)

Abbreviations: PR = pulmonary rehabilitation, PF = pulmonary function, FVC = forced vital capacity, FEV1 = 1 s forced expiratory volume, VO_2_ max = maximal oxygen consumption, MVV = maximal voluntary ventilation, VE = minute ventilation, VE/VCO_2_ = ventilatory equivalent for CO_2_, VLC = vital lung capacity PEF = peak expiratory flow, 6MWT = 6-min walk test, MOT = mean operating time, ERAS = enhanced recovery after surgery, MIP = maximal inspiratory pressure, CI = cardiac index, WR = work rate, CAT = Chronic Obstructive Pulmonary Disease Assessment Test, HRV = heart rate variability, SF-36 = 36-item short form survey, MFI-20 = multidimensional fatigue inventory, QoL = quality of life, EORTC-QLQ-C30 = European Organization for the Research and Treatment of Cancer Quality of Life Questionnaire, QF = quadriceps force, MRC = Medical Research Council, mMRC = modified Medical Research Council, SGRQ = St. George’s Respiratory Questionnaire, PPCs = postoperative pulmonary complications, FACT-L = functional assessment of cancer therapy—lung, SESPRM-LC = Self-Efficacy Scale for Postoperative Rehabilitation Management of Lung Cancer, HADS = hospital anxiety and depression scale, BODE = BMI, airflow obstruction, dyspnea, exercise capacity.
